# Crosstalk between Delta Opioid Receptor and Nerve Growth Factor Signaling Modulates Neuroprotection and Differentiation in Rodent Cell Models

**DOI:** 10.3390/ijms141021114

**Published:** 2013-10-21

**Authors:** Dwaipayan Sen, Michael Huchital, Yulong L. Chen

**Affiliations:** 1 Department of Biological Sciences, Binghamton University, the State University of New York at Binghamton, Binghamton, NY 13902, USA; E-Mails: dsen1@binghamton.edu (D.S.); mhuchital@gmail.com (M.H.); 2 The Center for Development and Behavioral Neurosciences, Binghamton University, the State University of New York at Binghamton, Binghamton, NY 13902, USA

**Keywords:** GPCR, delta opioid receptor, DADLE, NGF, Akt, MAPK, neuroprotection

## Abstract

Both opioid signaling and neurotrophic factor signaling have played an important role in neuroprotection and differentiation in the nervous system. Little is known about whether the crosstalk between these two signaling pathways will affect neuroprotection and differentiation. Previously, we found that nerve growth factor (NGF) could induce expression of the delta opioid receptor gene (*Oprd1*, *dor*), mainly through PI3K/Akt/NF-κB signaling in PC12h cells. In this study, using two NGF-responsive rodent cell model systems, PC12h cells and F11 cells, we found the delta opioid neuropeptide [d-Ala^2^, d-Leu^5^] enkephalin **(**DADLE)-mediated neuroprotective effect could be blocked by pharmacological reagents: the delta opioid antagonist naltrindole, PI3K inhibitor LY294002, MAPK inhibitor PD98059, and Trk inhibitor K252a, respectively. Western blot analysis revealed that DADLE activated both the PI3K/Akt and MAPK pathways in the two cell lines. siRNA *Oprd1* gene knockdown experiment showed that the upregulation of *NGF* mRNA level was inhibited with concomitant inhibition of the survival effects of DADLE in the both cell models. siRNA *Oprd1* gene knockdown also attenuated the DADLE-mediated neurite outgrowth in PC12h cells as well as phosphorylation of MAPK and Akt in PC12h and F11 cells, respectively. These data together strongly suggest that delta opioid peptide DADLE acts through the NGF-induced functional G protein-coupled Oprd1 to provide its neuroprotective and differentiating effects at least in part by regulating survival and differentiating MAPK and PI3K/Akt signaling pathways in NGF-responsive rodent neuronal cells.

## 1. Introduction

Opioids are a family of molecules that are composed of both opiate alkaloid compounds and peptides having opiate effects. Opioids have been used as painkillers for thousands of years. In addition, opioids are also involved in cell proliferation [1,2], angiogenesis [3], cell survival/neuroprotection [4–10], neurogenesis/neuronal differentiation [10–12], and the brain development [13]. There are endogenous peptides and three classic opioid receptors (mu, delta, and kappa, called Oprm1/MOR, Oprd1/DOR, and Oprk1/KOR, respectively) expressed in the peripheral and central nervous system. Genetic knockout studies clearly demonstrate that these three opioid receptors mediate effects of both endogenous and exogenous opioids on pain, reward, the development of opioid tolerance, addiction, and immune suppression [14]. The molecular mechanisms of opioid-mediated neuroprotection and differentiation are not quite clear. Because differentiation and neuroprotection are critically important in the development and maintenance of the nervous system [15], a better understanding of the molecular basis of the opioid-mediated effects should provide some insight into the neurological disorders associated with deregulation of these two processes in the brain.

The effects of opioids on neuroprotection and differentiation can be very complicated. Several factors may contribute to the complexity: (1) the opioid effects are usually cell-type dependent just as many other biochemical responses to drug treatment; (2) different opioid receptors mediate different downstream effectors, resulting in different phenotypical changes in cells; (3) many opioids may selectively act through each opioid receptor, but not exclusively; and (4) many opioids may also act through non-opioid receptors. All these factors together contribute to diverse observations that can be controversial [16]. The delta opioid signaling system has been shown to play a critical role in neuroprotection [7,10], neurogenesis [10], and neuronal differentiation [17]. Moreover, one of the opioids that have been consistently shown to have significant neuroprotective effect in many studies is synthetic peptide delta-selective agonist [d-Ala^2^, d-Leu^5^] enkephalin (DADLE) [18].

DADLE has been shown to have neuroprotective effects in cell and animal models [18]. DADLE protects against methamphetamine-induced dopaminergic terminal degeneration in the mouse brain, promotes functional effects of fetal rat mesencephalic dopaminergic cells, and reduces the neuronal damage caused by ischemia reperfusion [18]. Both DADLE and delta opioid antagonist treatment have shown that the endogenous delta opioid system may provide a self-protecting mechanism to improve neuron survival in ischemia-sensitive regions of the brain [6]. Moreover, DADLE also prolongs organ survival for transplantation and is proposed to use as an agent for protection of the peripheral and central nervous system [19]. A recent study further demonstrates that Oprd1 has a role not only in the induction of neural stem cells into differentiation, but also in protecting neuronal cells from apoptotic cell death induced by toxic agent H^2^O^2^[10]. More interestingly, both Oprm1 and Oprk1 have no effects on the induction of neural stem cell differentiation [10]. Similarly, delta opioid signaling offers neuroprotection in neocortical neurons, and both mu and kappa opioid signaling do not have the neuroprotective effect on the neurons [20]. However, the studies described above are mainly pharmacological studies, which have not completely resolved the question how the delta opioid signaling regulates neuroprotection and differentiation at the molecular level.

Neurotrophic factors are known to be critical for neuron survival and differentiation in the peripheral and central nervous system [21]. Delta opioid agonists are able to induce the expression of neurotrophins in varied situations and have anti-depressant effect [22,23]. Delta opioid agonist (+) BW373U86 treatment increases the brain-derived neurotrophic factor (*BDNF*) mRNA level in frontal cortex, hippocampus, basolateral amygdala, and endopiriform nucleus, while the delta antagonist naltrindole abolishes such increases in *BDNF* mRNA [23]. Chronic treatment of Swiss CD-1 mice with DADLE results in a significant increase in nerve growth factor (*NGF*) in the hippocampus and midbrain [24]. Previous studies have shown that NGF could induce *Oprd1* gene expression [25] through sustained activation of PI3K/Akt/NF-κB signaling-mediated epigenetic regulation mechanism in NGF-responsive PC12h cells [26–29]. It has been shown that DADLE has a neuroprotective effect in PC12 cells [18]. NGF is involved in both neuronal survival and differentiation [30]. Moreover, both NGF/TrkA and Oprd1 signaling are involved in MAPK and PI3K/Akt signaling pathways [31–33], which are known to mediate neuronal survival and differentiation [34,35]. Thus, the crosstalk between NGF signaling and DADLE/Oprd1 signaling may be a mechanism for the delta opioid signaling-mediated neuroprotective and differentiating effects both *in vitro* and *in vivo*. In this study, using a selective delta opioid agonist DADLE, we examined the causal effect of delta opioid signaling on neuroprotection and differentiation in the NGF-responsive PC12h cell line model. The causal effect of delta opioid receptor signaling on neuroprotection was then further confirmed in the F11 cell model, which was derived from rat dorsal root ganglion (DRG) neurons (from embryonic day 13–14 rats) with mouse neuroblastoma cells [36]. A series of siRNA experiments demonstrated that DADLE exerted these effects on NGF-responsive cells mainly through the crosstalk between NGF signaling and Oprd1 signaling at the mRNA level and through modulating both MAPK and PI3K/Akt signaling. As NGF-differentiated PC12 cells possess many features of the sympathetic neurons [37] and cAMP/NGF-differentiated F11 cells possess many features of the sensory DRG neurons [38,39], our results from this study may be applicable to other NGF-responsive neurons that express delta opioid receptors.

## 2. Results

### 2.1. DADLE Up-Regulated *NGF* mRNA in PC12h and F11 Cells

**Figure 1:**
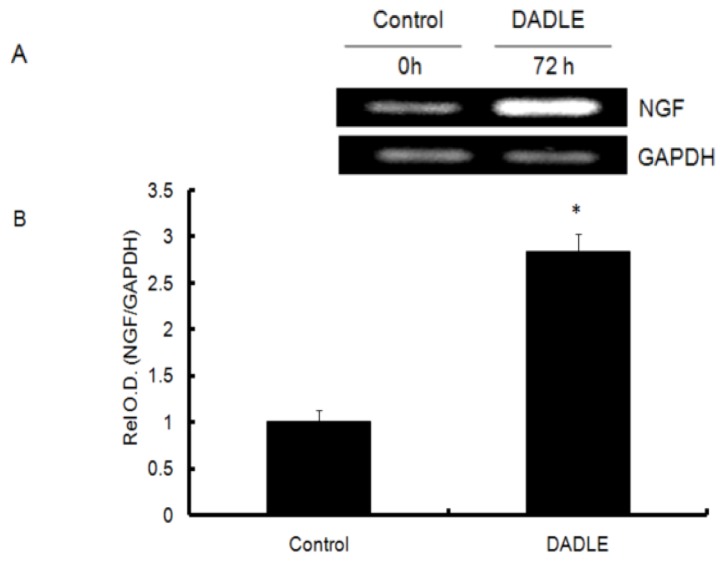
RT-PCR analysis of *NGF* expression in PC12h cells. PC12h cells were treated simultaneously with 100 ng/mL NGF and 10 nM DADLE for 72 h. After 72 h the total RNA was extracted and semi-quantitative RT-PCR was performed. (**A**) Induction of *NGF* mRNA after 72 h of DADLE treatment in NGF stimulated PC12h cells; (**B**) Relative optical density (Rel O.D.) of *NGF* RT-PCR product with or without DADLE treatment for 72 h. Rel O.D. of the untreated control was assigned to be unit one. Data are expressed as mean ± SEM of three independent experiments. * *p* < 0.05.

**Figure 2:**
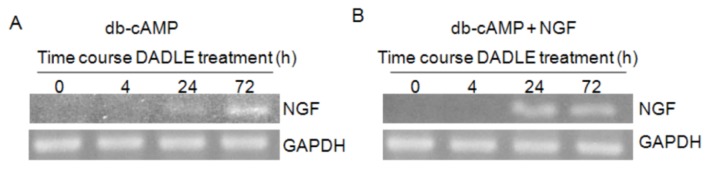

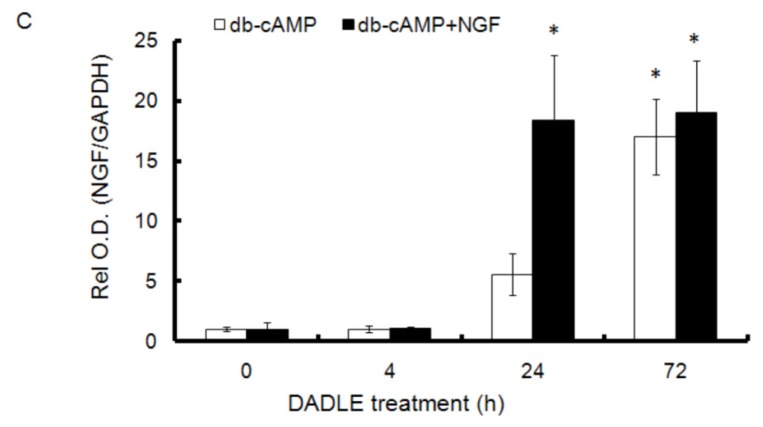
RT-PCR analysis of *NGF* expression in F11 cells. F11 cells were differentiated with 0.5 mM db-cAMP and with or without 50 ng/mL NGF for 72 h. DADLE (1 μM) was treated for varied times. After 72 h the total RNA was extracted and semi-quantitative RT-PCR was carried out. (**A**) Induction of *NGF* mRNA after 72 h DADLE treatment in F11 cells differentiated only in the presence of db-cAMP; (**B**) Induction of *NGF* mRNA after 72 h DADLE treatment in F11 cells differentiated in the presence of db-cAMP and NGF; (**C**) Data are expressed as mean ± SEM of three independent experiments. Relative optical density (Rel O.D.) of the *NGF* RT-PCR DNA band to that of the respective *GAPDH* band was normalized with Rel O.D. of the untreated control (DADLE treatment for 0 h) assigned to be unit one. * *p* < 0.05.

To investigate the effect of DADLE on *NGF* mRNA, PC12h cells were treated simultaneously with NGF and different doses (1.0–10,000 nM) of DADLE for 72 h. The controls were treated only with NGF. Total RNA was harvested after 72 h and semi-quantitative RT-PCR was carried out for rat *NGF*, with endogenous control *GAPDH* as described in Materials and Methods. Preliminary screening experiments showed that DADLE at 10 nM concentration markedly increased endogenous *NGF* expression in time-dependent manner reaching the peak expression at 72 h (Figure S1). A literature study has shown that DADLE has an antiapoptotic effect in nanomolar concentration in PC12 cells [40]. In the further experiments, cells were treated with DADLE (10 nM for PC12h cells, and 1 μM for F11 cells) for 72 h. Under these conditions, DADLE significantly up-regulated *NGF* mRNA levels in both PC12h and F11 cells (Figures 1 and 2). In addition, while the presence of differentiating agent db-cAMP increased *NGF* mRNA expression after 24 and 72 h in F11 cells, the presence of NGF in the medium enhanced *NGF* expression after 24 h of DADLE treatment (Figure 2). As NGF is known to be pro-survival in neuronal cells, these results indicate that enhanced expression of *NGF* may play a role in DADLE-enhanced neuronal survival in the two NGF-responsive cell lines.

### 2.2. Naltrindole, LY294002 (LY), and PD98059 (PD) Blocked DADLE-Increased Neurite Length and Number in Differentiating PC12h Cells

**Figure 3:**
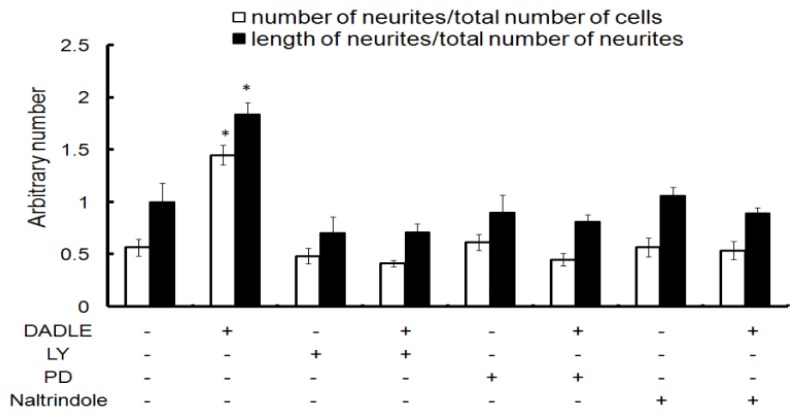
Effect of DADLE on the number of neurites normalized to the total cells and length of neurites normalized to the total neurites in PC12h cells. PC12h cells were cultured with 100 ng/mL NGF, with or without 10 nM DADLE (40,000 cells per 35 mm tissue culture dish). After 72 h of treatment, random pictures were taken (10 from each dish). Data are expressed as an average of two independent experiments in triplicate dishes for each treatment. Neurite length was measured using the Neuron J free software (http://www.imagescience.org/meijering/software/neuronj) [44] as described in Materials and Methods. * *p* < 0.05, compared DADLE-treated cells with untreated cells.

Naltrindole is a highly selective delta opioid receptor antagonist [41] and, in addition, an Akt signaling inhibitor [8]. LY compound is a PI3K inhibitor [42]; PD is a MAPK inhibitor [43]. To examine the effect of DADLE on NGF-induced differentiation of PC12h cells and the involvement of both PI3K/Akt and MAPK signaling in DADLE action, cells were treated with DADLE, naltrindole, LY and PD compounds as described in Materials and Methods. The cells were differentiated for 72 h. After 72 h, neurite length and number were measured as described in Materials and Methods. DADLE enhanced both neurite length (~1.8 fold) and number (~3 fold) in differentiating PC12h cells after 72 h (Figures 3 and Figure S2). The DADLE effects are consistent with that of increased *NGF* expression (Figure 1). Such an increase in endogenous *NGF* by DADLE may be part of the molecular mechanism underlying DADLE-mediated neuroprotection and differentiation. Indeed, naltrindole, LY, and PD all reduced the neuritogenic effect of DADLE (Figure 3). These results together suggest that DADLE may act through the delta opioid receptors to activate PI3K/AKt and the MAPK signal transduction pathways to mediate neurite outgrowth.

### 2.3. Naltrindole, LY, and PD Reduced the Neuroprotective Effect of DALDE on Cells in Serum-Free Medium

**Figure 4:**
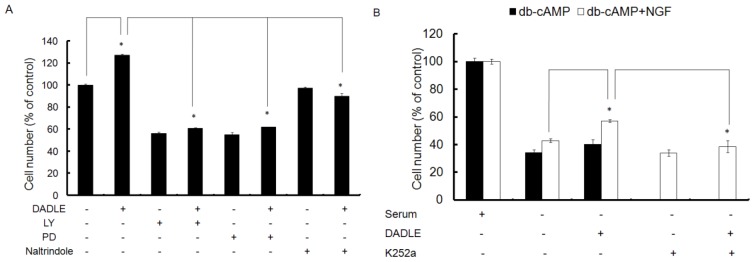
Survival effect of DADLE on PC12h and F11 cells as analyzed by MTT assay. (**A**) PC12h cells (10,000 cells) were plated in 96 well plates and differentiated for three days with 100 ng/mL NGF. After three days, the medium was removed, cells were washed and re-fed with serum free medium (without NGF) and treated with the respective compounds for another 48 h. MTT assay was performed as described in Materials and Methods; (**B**) F11 cells (10,000 cells/well) were plated in 96-well plates and differentiated for 72 h with 0.5 mM db-cAMP with or without 50 ng/mL NGF. Cells were also differentiated in the presence of db-cAMP, NGF and K252a. Cells were pretreated with 100 nM K252a for 30 min before addition of 50 ng/mL NGF. After 72 h of differentiation the medium was removed and cells were washed and re-fed with serum free medium with or without 1 μM DADLE for 48 h. As a positive control cells were continued to differentiate in db-cAMP in the presence or absence of NGF for a total of five days. After a total of five days in culture the cells were harvested and MTT assay was carried out as described in Materials and Methods. Data are expressed as mean ± SEM of three independent experiments done in six replicate wells for each treatment. * *p* < 0.05.

To investigate the effect of naltrindole, LY, and PD on DADLE-mediated neuronal survival, PC12h cells (10,000 cells/well) were plated in 96-well plates and differentiated for 72 h with 100 ng/mL NGF. Cell viability MTT assay was carried out as described in Materials and Methods. DADLE significantly enhanced survival of PC12h cells after 48 h in serum free medium by 27% (Figure 4A). The neuroprotective effect of DADLE in PC12h cells is consistent with those reported in literature [7,10,19,40]. When the cells were pretreated with 1 μM naltrindole, the neuroprotective effect of DADLE was inhibited, indicating that it may act through delta opioid signaling to promote its neuroprotective effect. Inhibiting both the PI3K and MAPK signaling pathways with LY and PD, respectively, also prevented the neuroprotetive effect of DADLE (Figure 4A). This result indicated that both MAPK and PI3K signaling pathways might be involved in the cell surviving effect of DADLE. It is noticeable that LY and PD alone significantly reduce cell survival (Figure 4A), indicating that these two signaling pathways are also critical for maintaining basal cell survival. This is most likely through an NGF-mediated autocrine and paracrine survival mechanism.

### 2.4. Trk Signaling Inhibitor K252a Blocked the Neuroprotetive Effect of DADLE on NGF- and cAMP-Differentiated F11 Cells

To further examine the effect of DADLE on neuronal survival in another cell model, F11 cells (10,000 cells/well) were plated in 96-well plates and differentiated for 72 h with 0.5 mM db-cAMP with or without 50 ng/mL NGF. Cells were also differentiated in the presence of db-cAMP, NGF and K252a with or without DADLE as described in Materials and Methods. K252a is a selective inhibitor of the tyrosine protein kinase activity of the trk family of oncogenes and neurotrophin receptors. As shown in Figure 4B, DADLE had a significant positive survival effect (34%) on F11 cells differentiated with db-cAMP only in the presence of NGF. K252a blocked the neuroprotective effect of DADLE (Figure 4B). In comparison with results from PC12h cells, it appears that DADLE has better neuroprotective effect (34% *vs*. 27%) in F11 cells. This difference is mainly due to the fact that F11 cells express a higher level of the Oprd1 receptor at both protein and mRNA levels. Figure S3 shows that in the presence of NGF the mouse *Oprd1* mRNA level was markedly higher in the presence of NGF than in the absence of NGF. This observation is consistent with the previous findings that NGF induces *Oprd1* expression in PC12h cells [25,29,45]. The results (Figures 4B and 3S) suggest that NGF may play a role in increasing DADLE-induced survival in F11 cells possibly by increasing the functional delta opioid receptors (Figure S3).

### 2.5. DADLE Enhanced Akt and MAPK Phosphorylation in PC12h and F11 Cells

**Figure 5:**
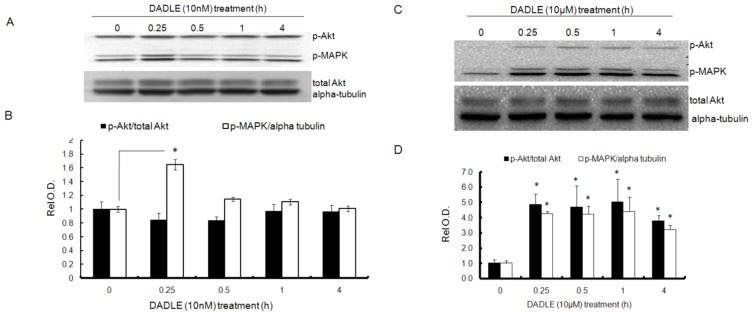
Effect of DADLE on phosphorylation of MAPK and Akt in PC12h cells: PC12h cells (0.8 million cells/60-mm dish) were differentiated with 100 ng/mL NGF for 72 h. The cells were re-fed once with 100 ng/mL NGF after 48 h of plating. After 72 h of differentiation, medium was removed from each dish and cells were re-fed with medium without NGF for 24 h. After 24 h of NGF deprivation, cells were treated with 10 nM or 10 μM DADLE for 0, 0.25, 0.5, 1, and 4 h. Cells were harvested and Western blotting was carried out for p-Akt (ser473), p-MAPK, total Akt and alpha tubulin as described in Materials and Methods. (**A**) DADLE (10 nM) induced phosphorylation of Akt and MAPK in PC12h cells; (**B**) Semi-quantification of phosphorylation of Akt and MAPK (10 nM DADLE); (**C**) DADLE (10 μM)-induced phosphorylation of Akt and MAPK; (**D**) Semi-quantification of phosphorylation of Akt and MAPK (10 μM DADLE). p-Akt was normalized to total Akt and p-MAPK to alpha tubulin. Relative O.D. was measured as described in Materials and Methods. Data are expressed as mean ± SEM of three independent experiments normalized to unit one for the 0 h treatment (control). * *p* < 0.05.

**Figure 6:**
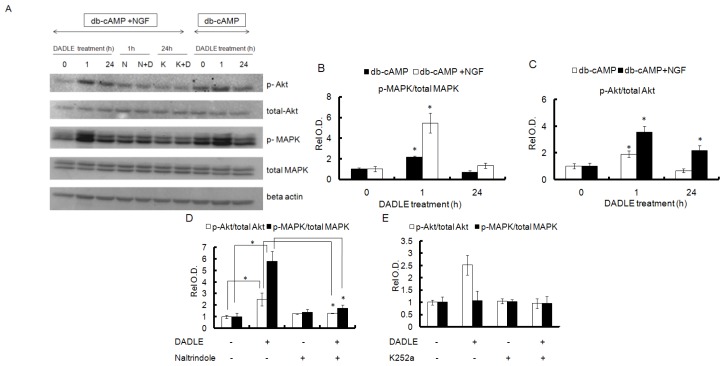
Naltrindole and K252 blocked DADLE-induced phosphorylation of MAPK and Akt in F11 cells: F11 cells were differentiated with 0.5 mM db-cAMP and with or without 50 ng/mL NGF for 72 h. Cells were treated with DADLE (1 μM) for 1 h and 24 h, respectively. The cells were pretreated with or without 100-μM naltrindole for 30 min before treating with or without DADLE for 1 h. Cells were also pretreated with 100 nM K252a for 30 min before treating with DADLE for 24 h. After a total of 72 h differentiation, the cells were harvested and Western blotting was carried out for p-Akt (ser 473), p-MAPK, total MAPK, total Akt and beta actin as described in Materials and Methods. (**A**) A representative immunoblot from cells differentiated in the presence of db-cAMP, K252 (K, 100 nM), and naltrindole (N, 100 μM); (**B**) Semi-quantification of p-MAPK normalized to total MAPK; (**C**) Semi-quantification of p-Akt normalized to total Akt. Data are expressed as mean ± SEM of three independent experiments normalized to unit 1 for the 0 h treatment (control); (**D**) Inhibition of phosphorylation of Akt and MAPK by naltrindole. Semi-quantified data from three independent runs are shown normalized to one for the control (no treatment); (**E**) Inhibition of the phosphorylation of Akt by K252a for 24 h. Semi-quantified average data from two independent runs are shown normalized to one for the control (no treatment). * *p* < 0.05.

NGF-induced sustained activation of PI3K/Akt signaling, resulting in upregulation of the *Oprd1* gene [29]. To evaluate whether DADLE-mediated neuroprotection and differentiation are regulated through the NGF-induced Oprd1 receptor, we examined the two Oprd1 downstream effectors, Akt and MAPK. As shown in Figure 5A,B, DADLE at a low dose (10 nM) activated the MAPK (p44/p42) pathway as indicated by the increased phosphorylation of the proteins after 0.25 h of DADLE treatment in PC12h cells. There was no change in the levels of phosphorylated Akt. As shown in Figure 5C,D, DADLE administered at a high dose (10 μM) activated both Akt and MAPK signaling significantly as early as 15 min. This response continued even after 24 h of treatment in PC12h cells. The data further demonstrated that the Oprd1 receptor was functional, indicating that DADLE mediated its neuroprotective effect through MAPK signaling at the low dose and through both MAKP and PI3K/Akt downstream signaling pathways at a higher dose. We further examined the effects of DADLE on MAPK and Akt phosphorylation in F11 cells. DADLE was treated in varied times and after 72 h of differentiation, the cells were harvested for immunoblot analysis as described in Materials and Methods. Figures 6 and S4 showed that in the presence of NGF, increased MAPK phosphorylation peaked at 1 h (Figures 6C and S4C), but Akt phosphorylation peaked at 1 h (Figures 6B and S4B) and dropped down at 8 h and then came up higher than the control and sustained for 48 h (Figure S4B). The nature of the biphasic phosphorylation of Akt is not understood at this time. Further experiments will be needed to confirm and to further evaluate such dynamic nature of phosphorylation of Akt. In the absence of NGF, phosphorylation of both MAPK and Akt peaked at 1 h (Figure 6). As shown in Figure S3, in the presence of NGF, F11 cells had the higher level of endogenous *Oprd1* mRNA. Here it was found that when F11 cells were differentiated in the presence of NGF, Akt was active after 24 h of DADLE treatment, but not MAPK. It is known that the *Oprd1* gene expression is mainly induced by sustained activation of the PI3K/Akt signaling in the presence of NGF in PC12h cells [29]. The current data in F11 cells also indicate that NGF may be required for increased *Oprd1* expression through sustained activation of PI3K/Akt pathway. In the absence of NGF, both MAPK and Akt had only one peak of increased phosphorylation after 1 h of DADLE treatment. This observation suggested that DADLE might have a greater and sustained positive effect on survival in the presence of NGF because of increased amount of *Oprd1* expression in F11 cells (Figure S3). Moreover, PCR analysis indicates that F11 cells have much higher level of *Oprd1* mRNA than PC12h cells. This may explain why we observed DADLE-enhanced phosphorylation of Akt at a dose of 10 μM in PC12h cells (Figure 5), but at a dose of 1 μM in F11 cells (Figure 6). Thus, our results indicated DADLE acted through the PI3K/Akt and MAPK pathways to induce its neuroprotective effect in both PC12h and F11 cells.

### 2.6. Naltrindole and K252a Reduced DADLE-Mediated Phosphorylation of MAPK and Akt in PC12h Cells and F11 Cells

**Figure 7:**
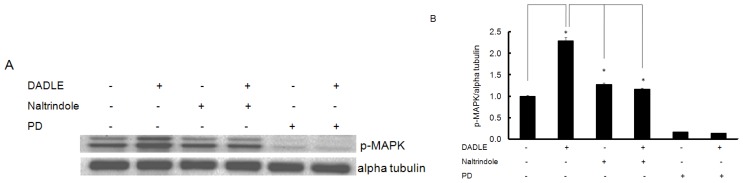
DADLE-increased phosphorylation of MAPK was blocked by naltrindole and PD in PC12h cell. PC12h cells (0.8 million cells/60-mm dish) were differentiated with 100 ng/mL NGF for 72 h. The cells were re-fed once with 100 ng/mL NGF after 48 h of plating. After 72 h of differentiation, medium was removed from each dish and cells were re-fed with NGF-free medium containing 0.1% DMSO for 24 h. After 24 h of NGF deprivation, the cells were pretreated with or without 10 μM PD and 1 μM naltrindole for 0.5 h before treating with or without 10 nM DADLE for 15 min. Cells were harvested and Western blotting was carried out for p-MAPK and alpha tubulin as the housekeeping protein. (**A**) Western blotting analysis of p-MAPK; (**B**) Semi-quantification of p-MAPK. p-MAPK was normalized to alpha tubulin. Relative O.D. was measured as described in Materials and Methods. Data are expressed as mean ± SEM of three independent experiments with all the data normalized to one for the control (no treatment). * *p* < 0.05.

To determine if Oprd1 was involved in the activation of Akt and MAPK by DADLE, PC12h and F11 cells were pretreated with naltrindole, K252a, and PD (only in PC12h cells) as described in Materials and Methods. Naltrindole inhibited the increase in phosphorylation of MAPK by DADLE in PC12h cells (Figure 7) and that of both Akt and MAPK in F11 cells (Figure 6), indicating that DADLE may act through the Oprd1 to induce its effect. We observed that F11 cells did not survive in 100 μM of naltrindole for 24 h. This is most likely because high concentration of naltrindole blocked the basal survival PI3K/Akt signaling [8,46], resulting in apoptotic death of F11 cells. To see if the increase in Akt phosphorylation after 24 h of DADLE treatment on F11 cells differentiated in the presence of NGF is specific to the presence of NGF, the cells were treated with 100 nM K252a before adding DADLE for 24 h of treatment. Under this condition, K252a inhibited increased phosphorylation of Akt after 24 h of DADLE treatment (Figure 6), indicating that the presence of NGF in the medium indeed plays a role in the late phase activation of Akt by DADLE in F11 cells.

### 2.7. Knockdown of *Oprd1* Using siRNA in PC12h and F11 Cells

**Figure 8:**
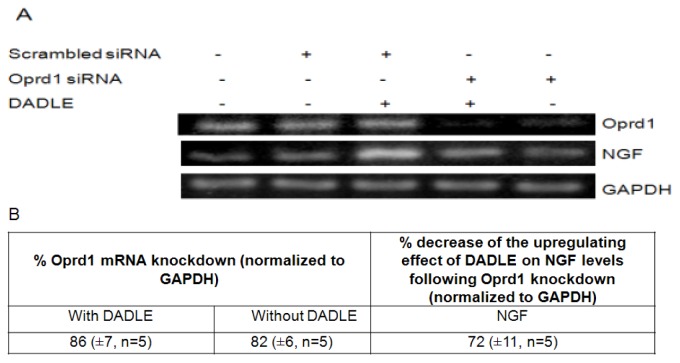
RT-PCR analysis of *NGF* expression in PC12h cells after 72 h DADLE treatment following knockdown of the *Oprd1* gene. Following transfection with *Oprd1* (*dor*) siRNA (cocktail of 3 siRNAs, 500 nM each) and scrambled siRNA (1.5 μM), 0.5 or 0.8 million cells/60-mm dishes were differentiated with 100 ng/mL NGF for 72 h in medium supplemented with or without 10 nM DADLE. After 72 h cells were harvested for total RNA isolation. Reverse transcription was carried out with 2 μg of total RNA for each sample followed by PCR. (**A**) The PCR products were run on a 3% agarose gel and stained with ethidium bromide; (**B**) Table showing the percent decreases of mRNA levels of *NGF* following *Oprd1* knockdown. Data are expressed as mean ± SEM of five independent experiments.

**Figure 9:**
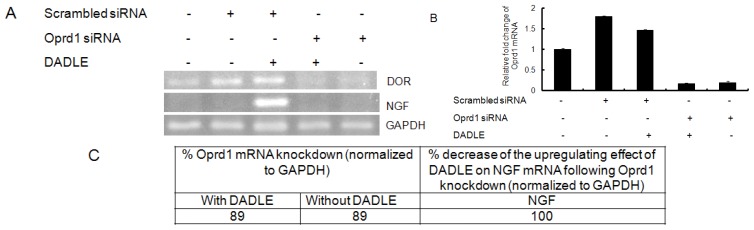
Quantitative-PCR analysis of *NGF* expression in F11 cells after 72 h DADLE treatment following knockdown of the *Oprd1* gene. Following transfection with *Oprd1* siRNA (cocktail of three siRNAs, 500 nM each) and scrambled siRNA (1.5 μM), 0.5 million cells/60-mm dishes were differentiated with 0.5 mM db-cAMP and 50 ng/mL NGF for 72 h in medium supplemented with or without 1 μM DADLE. After 72 h, cells were harvested for total RNA isolation. Reverse transcription was performed with 2 μg of total RNA for each sample. PCR was performed for rat *NGF*, mouse *Oprd1* and *GAPDH*. (**A**) Representative semi-quantitative PCR products that were run on a 3% agarose gel and stained with ethidium bromide; (**B**) Relative fold change of mouse *Oprd1* as compared to *GAPDH* obtained from quantitative PCR. The data is normalized to one for the no siRNA treatment. Quantitative PCR was run using cDNA samples pooled from two independent runs. Data is from one PCR run done in replicates of four for each treatment; (**C**) Table showing the percent decrease of mRNA levels of *NGF* and *Oprd1* following *Oprd1* knockdown.

**Figure 10:**
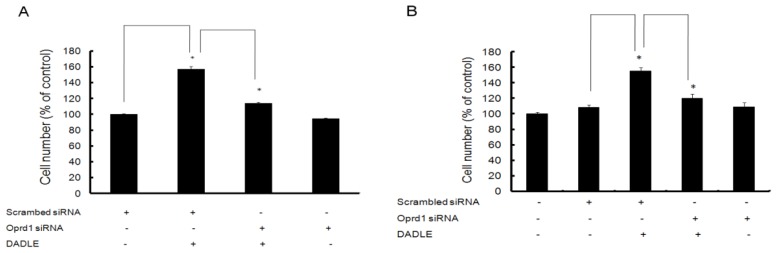
The positive survival effect of DADLE was inhibited by knocking down the *Oprd1* gene in PC12h cells and F11 cells, respectively. Following transfection with *Oprd1* siRNA (cocktail of three siRNAs, 500 nM each) and scrambled siRNA (1.5 μM), 10,000 cells were plated in each well of a 96-well plate containing 100 μL of pre-warmed medium supplemented with 100 ng/mL NGF without DADLE. After three days, medium was removed from each well and cells were washed once with serum free medium. The cells were re-fed with serum free medium (without NGF) with or without 10 nM DADLE (**A**) or 1 μM DADLE (**B**) for 48 h. After 48 h, MTT assay was performed as described in Materials and Methods. Data are expressed as mean ± SEM of three independent experiments for **A** and two independent runs for **B** done in six replicate wells for each treatment. * *p* < 0.05.

To elucidate the role of Oprd1 in DADLE-induced expression of endogenous neurotrophins like *NGF*, siRNA was used to knockdown the *Oprd1* gene expression in both PC12h and F11 cells as described in Materials and Methods. As shown in Figure 8, knocking down the *Oprd1* mRNA inhibited DADLE-mediated upregulation of *NGF*, suggesting that DADLE acted through the functional Oprd1 to increase the expression of the survival gene *NGF* in PC12h cells. As shown in Figures 2 and 9, under the chosen PCR conditions, both semi-quantitative and quantitative PCR failed to detect *NGF* mRNA in F11 cells when the cells were not treated with DADLE. After DADLE treatment, *NGF* expression greatly increased which was completely lost following knockdown of *Oprd1* gene by 89%. This observation suggested that DADLE acted through the Oprd1 to increase the expression of *NGF* gene in F11 cells as well. Figures 8 and 9 show that following *Oprd1* knockdown, the upregulating effect of DADLE on endogenous neurotrophins like *NGF* was inhibited in PC12h and F11 cells. It was not clear if this inhibition had any effect on DADLE-mediated neuroprotection on these cell lines. To establish if the lack of Oprd1 followed by decrease in *NGF* up regulation by DADLE had any effect on DADLE-mediated neuroprotection on PC12h and F11 cells, siRNA was used to knockdown the *Oprd1* and survival assay was carried out as described in Materials and Methods. Figure 10 shows that in both PC12h and F11 cells, following *Oprd1* knockdown, the positive survival effect of DADLE was inhibited by 75%–80%, indicating that DADLE acted through the Oprd1 to promote its neuroprotective effect.

**Figure 11:**
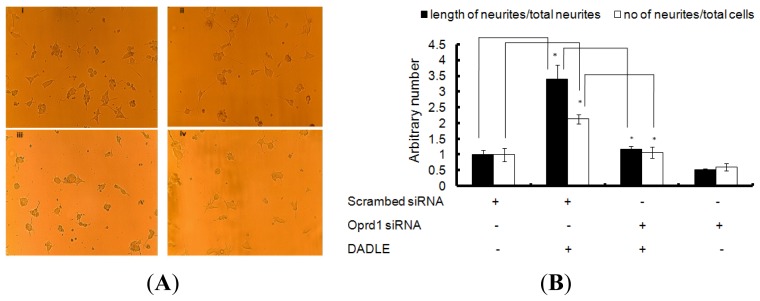
Inhibition of the positive neuritogenesis effect of DADLE by *Oprd1* knockdown in PC12 cells. Following transfection with *Oprd1* siRNA (cocktail of three siRNAs, 500 nM each) and scrambled siRNA (1.5 μM), 0.5 or 0.8 million cells/60-mm dishes were differentiated with 100 ng/mL NGF for 72 h in medium supplemented with or without DADLE. Before the cells were harvested, five to six random frames were captured from each dish by VistaVision USB camera (VWR). Neurite length was measured using the Neuron J software by semi-automatically tracing the neurites using a cutoff of 22 pixels as described in the Materials and Methods. (**A**) Representative pictures of differentiating PC12h cells transfected with scrambled siRNA without DADLE treatment (panel i), scrambled siRNA without DADLE treatment (panel ii), *Oprd1* siRNA with DADLE treatment (panel iii) and *Oprd1* siRNA without DADLE treatment (panel iv); (**B**) Data are expressed as mean ± SEM of three independent experiments normalized to one for the control (scrambled siRNA without DADLE). * *p* < 0.05.

To determine if Oprd1 is essential in DADLE-induced neuritogenesis, siRNA was used to knockdown the *Oprd1* gene in PC12h cells. Following knockdown, the length and number of neurites in differentiating PC12h in the presence or absence of DADLE were measured as described in Materials and Methods. *Oprd1* siRNA treatment led to marked reduction in both DADLE-mediated neurite length and number as shown in (Figure 11). Simultaneous inhibition of the positive neuritogenesis (~90%) (Figure 11) and survival effect of DADLE (75%–80%) following *Oprd1* knockdown (Figure 10) indicated that DADLE acted through the Oprd1. In addition, these data indicated that the neuroprotective effect of DADLE on PC12h and F11 cells might be in part induced by up-regulation of endogenous neurotrophins like *NGF* following DADLE treatment.

**Figure 12:**
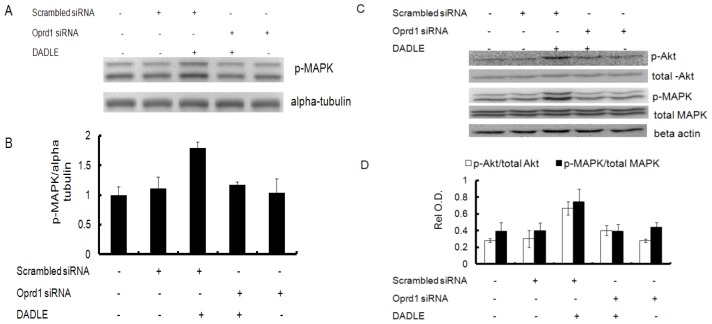
The increased phosphorylation of MAPK and Akt after DADLE treatment was inhibited following knockdown of the *Oprd1* gene in PC12h and F11 cells. For the PC12h cell experiments, following transfection with *Oprd1* siRNA (cocktail of three siRNAs, 500 nM each) and scrambled siRNA (1.5 μM), 0.2 million cells/60-mm dish was differentiated with 100 ng/mL NGF for 72 h. The cells were re-fed once with 100 ng/mL NGF after 48 h of plating. After 72 h, the medium was removed and cells were re-fed with 2 mL of medium without NGF and serum. After 24 h of NGF deprivation, cells were treated with or without (vehicle only) 10 nM DADLE for 15 min. After 15 min, total lysates were harvested for each condition and western blotting was carried out for p-MAPK with alpha tubulin as the loading control. For the F11 cell experiments, F11 cells (0.2 million cells) were plated in each of 60-mm dishes after transfection containing 2 mL of 0.1% serum medium, 0.5 mM db-cAMP, 50 ng/mL NGF treated with or without 1 μM DADLE for 1 h. (**A**) Immunoblot of p-MAPK; (**B**) Semi-quantification of p-MAPK. p-MAPK was normalized to alpha tubulin in PC12h cells; (**C**) Representative immunoblots for p-Akt, p-MAPK, total-Akt, total-MAPK and beta actin in F11 cells; (**D**) Semi-quantification of p-Akt and p-MAPK in F11 cells. p-Akt was normalized to total Akt and p-MAPK was normalized to beta actin. Relative O.D. was measured as described in Materials and Methods. Data are expressed as average from two independent runs with each cell line.

To investigate if DADLE activated MAPK and Akt in an Oprd1-dependent manner, PC12h and F11 cells were transfected with siRNA to knockdown the *Oprd1* mRNA as described in Materials and Methods. As shown in Figure 12A,B, following *Oprd1* knockdown in PC12h cells, the increased phosphorylation of MAPK by DADLE was inhibited, suggesting that Oprd1 is involved in DADLE-induced neuroprotection. Following Oprd1 knockdown in F11 cells, the increased phosphorylation of MAPK and Akt by DADLE was inhibited (Figure 12C,D), suggesting that Oprd1 signaling is involved in DADLE-induced activation of both MAPK and PI3K/Akt pathways in F11 cells.

## 3. Discussion

Opioids have been used for painkillers for thousands of years. Besides the analgesic effects, opioid compounds have many non-analgesic effects. Among these non-analgesic effects are neuroprotection and differentiation. Both neuroprotection and differentiation are associated with neuronal development. Chronic exposure to opioids can lead to alterations in brain structure and functional connectivity in humans [47] and in neuronal structure in rodents [48]. Although there are some conflicting observations for general opioid-mediated neuroprotection and differentiation *in vivo* and *in vitro* ([2,16] and the references cited therein), recent studies have demonstrated that delta opioid signaling-mediated neuroprotection and differentiation appeared to be relatively consistent in different cell-type models [7,9,10,16,17]. Such non-analgesic cellular effects are important because delta opioid-mediated neuroprotection and differentiation have potential clinical applications in areas such as organ transplantation, treatment for neurodegenerative disorders [9], and mood disorders [49]. Depression is associated with neuronal development and Oprd1 signaling [49]. Furthermore, delta opioid agonists have shown to have effects similar to those of antidepressants by inducing neurotrophic factor *BDNF* in several brain regions [22]. Thus, it is biologically and clinically important to understand how the delta opioids mediate neuroprotection and differentiation. However, the molecular mechanisms underlying delta opioid-mediated neuroprotection and differentiation are not well understood. In the current study, using two NGF-responsive cell models, the PC12h cell line and the F-11 cell line, we examined an opioid/NGF crosstalk-mediated neuroprotection hypothesis. We found that the crosstalk between NGF signaling and Oprd1 signaling may play a major role in the neuroprotective effect of DADLE in these rodent cells. In addition, in PC12h cells, the neurite outgrowth and numbers of neurite per cell are NGF-dependent, and DADLE affects both neurite length and numbers. Since these experiments were carried out in the presence of NGF with or without DADLE, the net effects observed were likely from DADLE-mediated endogenous NGF, MAPK, and PI3K/Akt survival and differentiating signaling. Our pharmacological, biochemical, and siRNA studies together with cell morphological studies indicate that DADLE enhances *NGF* gene expression, leads to more sustained activation of NGF signaling through Oprd1-mediated activation of both MAPK and PI3K/Akt signaling pathways, and eventually results in enhanced neuronal survival and modulation of neurite outgrowth and number of neurites in NGF-responsive cells.

Various pharmacological studies have shown that there are two delta opioid receptors: delta-1 opioid receptor and delta-2 opioid receptor [50]. These two subtypes sometimes also have opposing pharmacological activities [51]. However, the delta-2 opioid receptor gene has yet to be identified. Opioid receptor dimerization has been proposed [52] and shown to be responsible for the pharmacological activity of the delta-2 opioid receptor [51]. Previous pharmacological studies have shown that the delta-2 opioid receptor may be involved in neurite outgrowth [53,54]. Moreover, the high doses and low doses of opioids also have opposite effects on neurite formation in PC12 cells [55]. Our result from DADLE-mediated neuronal differentiation (Figures 3 and 11) is consistent with the observation by others in neuronal progenitor AF5 cells [17], as well as in mouse forebrain neural stem cells [10]. Our *Oprd1* (*dor*) siRNA knockdown studies clearly indicated that DADLE acted through Oprd1 to regulate both neuroprotection (Figure 10) and neuronal differentiation (Figure 11). Thus, our results together with others suggest that the pharmacological activity of the delta opioid receptor subtypes and opposite dose-responses observed by other researchers likely result from either the homodimer, heterodimer, oligomers formed by Oprd1 with other opioid receptors [51,56] or by Oprd1 with other non-classic opioid receptor interaction such as the cannabinoid receptor [57].

Brain development is regulated by apoptotic cell death and differentiation. Neurotrophic factors modulate both neuronal survival and differentiation through their respective receptor-mediated MAPK and PI3K/Akt signaling [58]. The neocortex and hippocampus are the brain regions where *NGF* is expressed both during development and in adults [58]. It has been known that opioids affect brain development [59]. Chronic opioid exposure affects not only the developing brain [60], but also the adult brain [47,61]. The delta opioid receptor plays an important role in opioid-mediated neuronal survival and differentiation [2,16]. The results from our current studies of two rodent cell models clearly demonstrated that delta opioid DADLE acted through Oprd1 to activate the two main downstream PI3K/Akt and MAPK signaling pathways regulating neuronal survival and neuronal differentiation. This is also consistent with the literature observations that NGF-induced and DADLE-induced activation of MAPK signaling is involved in neurite outgrowth in PC12 cells [62]. One possible regulatory mechanism is the regulation of expression of critical survival and differentiation-associated genes such *NGF*, *BDNF*, *GDNF*, and other neurotrophic factors in an autocrine and paracrine manner [63]. In PC12h cells, because both neuronal survival and differentiation are mainly dependent upon NGF signaling, we observed that *Oprd1* knockdown led to the down-regulation of *NGF* expression, resulting in a reduction in both cell survival and differentiation as expected (Figures 10 and 11). Our preliminary data also showed that DADLE could induce *BDNF* and *GDNF* expression through Oprd1 signaling (unpublished data). Since it has been known that there is no BNDF receptor TrkB expressed in PC12 cells [64], induction of *BDNF* might not contribute to the DADLE-mediated cell survival and neuronal differentiation in PC12h cell. As BDNF and GDNF are important for cell survival in other cell types [65], DADLE may act in similar ways as represented in the two cell lines to mediate neuronal survival through regulation of *BDNF* and *GDNF* or other neurotrophic factors in other types of neuronal cells. It is clear that the crosstalk between DADLE/Oprd1 signaling and NGF/TrkA signaling mediates both neuronal survival and differentiation in the NGF-responsive PC12h cells. NGF is also required for survival of nociceptive DRG neurons during embryonic development [66]. Moreover, we also observed DADLE/Oprd1 signaling is involved in F11 cell survival (Figures 4B and 10B). Taken together, it is very tempting to speculate that the crosstalk between delta opioid signaling and neurotrophic factor signaling may be one mechanism underlying delta opioid-mediated neuroprotection and differentiation in different cell-types of neurons expressing neurotrophic factors. Further evaluation of the mechanisms of action of opioids on the different neurotrophic factor-dependent cell types will be needed to have a more completed mechanism. Nonetheless, our current study has shed some light on this direction.

Regulation of neurotrophic factors has been an intense drug discovery effort because of the important roles of neurotrophic factors in the maintenance and the development of the peripheral and central nervous system [67]. Pharmacological control of neurotrophic factor gene expression has been hypothesized to provide potential therapeutic interventions for diverse neurodegenerative disorders [68] such as Parkinson’s and Alzheimer’s diseases, both of which are associated with altered expression of neurotrophic factors during disease progression [69,70]. Our results from NGF-responsive rat neuron models show that DADLE regulates NGF signaling through Oprd1 signaling to prevent serum-free induced cell death (Figures 4 and 10). Our results are consistent with the earlier literature reports that delta opioids protect neurons from cell death in Parkinson’s, Alzhermer’s, and stoke models [7,9,71]. Thus, our current study together with the results from literature suggests that delta opioid agonists may be a potential lead compound for developing the NGF signaling activators and such NGF activators may be further developed for the prevention and treatment of NGF or other neurotrophic factor-dependent neurological disorders.

In conclusion, this study has clearly shown that NGF-induced G-protein coupled Oprd1 (DOR) in NGF-responsive cells are functional and the delta opioid signaling may mediate neuroprotection and differentiation at least in part through regulating *NGF* gene expression by activating MAPK and PI3K/Akt survival signaling in NGF-responsive cells.

## 4. Materials and Methods

### 4.1. Reagents

[d-Ala^2^, d-Leu^5^]-Enkephalin acetate salt (DADLE), dibutyryl cAMP sodium salt (db-cAMP), and naltrindole (NTI) from Sigma. LY294002 (LY), PD98059 (PD) and K252a were purchased from CalbioChem. (La Jolla, CA, USA). Effectene^®^ transfection reagent was purchased from Qiagen, Valencia, CA, USA (Catalog #301425). MTT assay kit was purchased from Promega (Madison, WI, USA). Nerve growth factor (2.5S) (NGF) was purchased from Harlan Bioproducts for Science, Inc. (Indianapolis, IN, USA).

### 4.2. Cell Culture

Seed PC12h (a subclone of PC12) cells [45], were generous gifts from Dr. Hiroshi Hatanaka in Japan obtained through Drs. Horace H. Loh and Ping Yee Law at University of Minnesota. PC12h cells were grown and maintained in 1:1 Ham’s F-12 medium/Dulbecco’s modified Eagle’s medium (F-12/DMEM) containing 5% horse serum and 5% calf serum (medium A). Seed F11 cells [36] were generous gift from Dr. Richard Miller at Northwestern University. F11 cells were grown and maintained in DMEM containing 10% fetal bovine serum (FBS) (medium B). Cells were maintained in a humidified 37 °C, 5% CO^2^ incubator. F11 cells were differentiated with 0.5 mM db-cAMP in 0.1% serum containing medium.

### 4.3. DADLE Treatment

For PC12h cell experiments, PC12h cells (0.8 million cells per 60-mm dish) were plated in 2 mL of medium A. After 24 h of plating, the medium was removed and the cells were treated simultaneously with 100 ng/mL NGF and with or without DADLE in 2 mL of 0.1% serum medium for 72 h. After 48 h, the cells were re-fed with 0.5 mL of 0.1% serum containing medium supplemented with 100 ng/mL NGF and with or without DADLE. For F11 cell experiments, F11 cells (0.3 million cells/60 mm dish) were plated in 2 mL of DMEM containing 10% FBS. After 24 h of plating, the medium was removed and the cells were treated simultaneously with 0.5 mM db-cAMP, with or without 50 ng/mL NGF and with or without DADLE in 2 mL of 0.1% serum medium for 72 h. After 72 h the total RNA was harvested. All treatments were done in duplicate or triplicate dishes.

### 4.4. Total RNA Extraction

Total RNA was harvested with the Tri Reagent RT kit according to the manufacturer’s protocol (Molecular Research Center, Inc., Cincinnati, OH, USA, Catalog # RT111). Purity of RNA was determined by spectrophotometric analysis at 260 nm and 280 nm.

### 4.5. Reverse Transcription and PCR

Reverse Transcription was performed using the High Capacity cDNA Reverse Transcription Kit according to the manufacturer’s protocol (Applied Biosystems, Foster city, CA, USA, Catalog #4368814). Total of 2 μg RNA was used for reverse transcription for each sample in a total volume of 20 μL. PCR was carried out using the Platinum Blue PCR Supermix (Invitrogen, Carlsbad, CA USA; Catalog # 12580-015). A total reaction volume of 25 μL was used with 2 μL of cDNA and 0.4 μM each of forward and reverse primers. The primers used for PCR are given in Table 1. All primers are designed using the OligoAnalyzer 3.1 (Integrated DNA Technologies, Coralville, Iowa, USA) with the default parameters. The cut off value of ΔG for hairpin, self and hetero dimmer formation of the primers was −6 kcal/mole. *NGF* and *Oprd1* PCR products are confirmed by sequencing (Table S1).

### 4.6. Western Blot

For PC12h cell experiments, PC12h cells (0.8 million/60-mm tissue culture dish) were plated in 2 mL of medium A. After 24 h of plating, medium in each dish was replaced by 2 mL of 1:1 F-12/DMEM containing 0.1% serum and 100 ng/mL NGF. The cells were differentiated with 100 ng/mL NGF for 72 h. The cells were re-fed once with 0.5 mL of 0.1% serum medium containing 100 ng/mL NGF, after 48 h of plating. After 72 h of differentiation, medium was removed from each dish and supplemented with medium without NGF for further 24 h. After 24 h of NGF deprivation, cells were treated with DADLE in varied times. For experiments with naltrindole and PD, after 24 h of NGF deprivation (following 72 h of differentiation with 100 ng/mL NGF), the cells were pretreated with or without 10 μM PD and 1 μM naltrindole for 30 min before treating them with or without DADLE for 15 min. For F11 cell experiments, F11 cells (0.3 million/60-mm tissue culture dish) were plated in 2 mL of medium B. After 24 h of plating, medium in each dish was replaced by 2 mL of DMEM containing 0.1% FBS, 0.5 mM db-cAMP, and with or without 50 ng/mL NGF. The cells were administered with 1 μM DADLE, 100 μM naltrindole and 100 nM K252a for different time points. Cells were pretreated for 30 min with K252a and naltrindole before DADLE and NGF were added, respectively. After 72 h of differentiation, the cells were harvested for the lysates and immunoblotting was carried out according to the manufacturer’s protocol (Cell signaling, Danvers, MA, USA) for phosphorylated (p)-Akt (ser473), total Akt, phosphorylated (p) MAPK (p-44/42), total MAPK using beta actin or tubulin as the comparison for sample loading control. Either 10 μL or 10 μg of each sample was loaded.

### 4.7. MTT Assay

For PC12h cell experiments, PC12h (10,000 cells) were plated in 96 well plates in 100 μL of medium A per well. After 24 h of plating, medium in each well was replaced with 100 μL of 0.1% serum medium supplemented with 100 ng/mL NGF. After 48 h, the cells were re-fed with 50 μL of the same fresh medium. The cells were differentiated for 3 days. After 3 days the serum containing media was removed and washed with serum free media once. The cells were then fed with 100 μL of serum free media (without NGF) with or without 10 nM DADLE, pretreated for 30–40 min with 1 μM naltrindole or 10 μM of LY or PD before DADLE was added for 48 h. For F11 cell experiments, F11 cells (10,000 cells per well) were plated in 96 well plates and differentiated for 72 h in the presence of 0.5 mM db-cAMP with or without 50 ng/mL NGF. Cells were also differentiated in the presence of db-cAMP, NGF and K252a with or without DADLE. Cells were pretreated with 100 nM K252a for 30 min before addition of 50 ng/mL NGF. After 72 h, the medium was removed and cells were washed with serum free medium and incubated with serum free medium with or without 1 μM DADLE for 48 h. As a positive control, cells were differentiated in db-cAMP in the presence or absence to NGF for a total of 5 days. After a total of 5 days in culture the cells were harvested and MTT assay was carried out according to the manufacturer’s protocol (Promega, Madison, WI, USA).

### 4.8. Morphological Study

PC12h cells were plated (40,000 cells per 35-mm tissue culture dish) in 2 mL of medium A. After 24 h of plating, medium in each dish was replaced by 2 mL of 0.1% serum medium containing 100 ng/mL NGF, with or without (control) 10 nM DADLE. Cells were also treated with 10 μM LY or 10 μM PD or 1 μM naltrindole. Cells were re-fed with NGF and their respective compounds after 48 h of plating. After total 72 h of treatment, random pictures were taken (10 per dish for Figure 3 and 5 or 6 per dish for Figure 11) by VistaVision USB camera (VWR, Radnor, PA, USA). Neurite length was traced semi-automatically from the start of the cell body to the end using the default parameters in the Neuron J 1.4.1 free software (www.imagescience.org/meijering/software/neuronj) [44]. Each branched neurite was considered separately in the measurement. A cut off length of 22 pixels was used—22 pixels was the average size of a PC12h cell as determined by averaging the maximum and minimum lengths of 200 cells with Neuron J 1.4.1 free software (see above).

### 4.9. RNA Interference

Cells (3 million/100 μL nucleofactor reagent) were transfected for 72 h with a cocktail of 3 siRNAs (500 nM each) against the rat *Oprd1* gene (PC12 cells) or mouse *Oprd1* gene (F11 cells) (Tables 2 and 3) and with 1.5 μM scrambled siRNA (negative control) using the program A029 for PC12 cells and X023 for F11 cells in the Amaxa^®^ Nucleofactor^®^ II device (Lonza, Walkersville, MD, USA). There was no visible change in cell morphology after transfection with transfection efficiency of around 100% (data not shown). After transfection, 500 μL of pre-warmed differentiating medium were added to each cuvette for the respective cell lines. Cells (0.5 or 0.8 million) were then aliquoted into 60-mm dishes containing 2 mL of pre-warmed (37 °C) medium supplemented with or without DADLE (10 nM and 1 μM DADLE for PC12h and F11 cells, respectively). An “untransfected” control was also included.

#### 4.9.1. Total RNA Isolation (Tri Reagent RT, MRC)

The experimental conditions were: (a) cells untransfected without DADLE as negative control; (b) transfected with *Oprd1* siRNA and DADLE; (c) transfected with *Oprd1* siRNA without DADLE; (d) transfected with scrambled siRNA and DADLE; and (e) transfected with scrambled siRNA without DADLE. After 72 h cells were harvested for total RNA isolation. Reverse transcription was carried out with 2 μg of total RNA for each sample in a total volume of 20 μL as described above. PCR was performed for rat *NGF* (36 cycles) rat *Oprd1* (36 cycles), mouse *Oprd1* (27 cycles) and *GAPDH* (23 cycles).

#### 4.9.2. Morphological Study

Before harvesting the cells for total RNA, 5 to 6 random frames were captured from each dish by VistaVision USB camera (VWR, Radnor, PA, USA). Neurite length was measured using the Neuron J free software (www.imagescience.org/meijering/software/neuronj) [44]) as described above.

#### 4.9.3. Western Blotting Analysis of siRNA-Treated Samples

The conditions for Western blotting analysis were: (a) without any transfection; (b) transfected with *Oprd1* siRNA; and (c) transfected with scrambled siRNA. PC12h cells (0.2 million cells) were plated in each of 60-mm dish for each treatment after transfection. After 48 h of transfection the cells were re-fed with 0.5 mL of 0.1% serum medium containing 100 ng/mL NGF. After 72 h of transfection, the medium was removed and cells were fed with 2 mL of medium without NGF. After 24 h of NGF deprivation, cells were treated with or without (vehicle only) 10 nM DADLE for 15min. After 15 min, cell lysates were obtained for each treatment condition and Western blotting was carried out for p-MAPK with alpha tubulin as the loading control as described previously. For the F11 cell experiment, F11 cells (0.2 million cells) were plated in each of the 60-mm dishes after transfection in 2 mL of 0.1% serum medium containing 0.5 mM db-cAMP and 50 ng/mL NGF for 72 h and treated with or without 1 μM DADLE for 1 h. The cells were harvested and immunoblotting was carried out according to the manufacturer’s protocol (Cell signaling, Danvers, MA, USA, for p-Akt (Ser473), total Akt, p-MAPK, total MAPK using beta actin as the comparison for sample loading control. Each sample (10 μL) was loaded into each well.

#### 4.9.4. Survival Assay

PC12h or F11 cells (10,000 cells from the remainder of the cell suspension after transfection) were plated in each well of a 96-well plate in 100 μL of pre-warmed medium containing 100 ng/mL of NGF for PC12h cells or 50 ng/mL of NGF with 0.5 mM db-cAMP for F11 cells, respectively. After 72 h, medium was removed from each well and washed once with serum free medium. The cells were re-fed with serum- and NGF-free medium (100 μL/well) with or without 10 nM DADLE in PC12h cells and with or without 1 μM DADLE in F11 cells for 48 h, respectively. MTT assay was performed as described above.

#### 4.9.5. Quantitative PCR

siRNA was used to knockdown the mouse *Oprd1* gene in F11 cells as described above. cDNA samples from 2 independent experiments were pooled together to run the PCR. PCR for each treatment were performed in 4 replicates using the iQ5 multicolour real time PCR detection system (Bio-Rad, Hercules, CA, USA). The protocol used for running mouse *Oprd1* and *GAPDH* using 1QTM SYBR Green Supermix (Bio-Rad, Hercules, CA, USA) was Cycle 1: (1X) Step 1: 95.0 °C for 3 min. Cycle 2: (45X), Step 1: 95.0 °C for 10 s. Step 2: 60.0 °C for 30 s. Cycle 3: (1X) Step 1: 95.0 °C for 1 min. Cycle 4: (1X) Step 1: 55.0 °C for 1 min. Cycle 5: (81X) Step 1: 55.0–95.0 °C for 15 s. The protocol for running rat *NGF* and *GAPDH* using TaqMan gene expression master mix (Applied Biosystems, Grand Island, NY, USA Catalog # 4369016) was Cycle 1: (1X) Step 1: 50.0 °C for 2 min. Cycle 2: (1X) Step1: 95.0 °C for 10 min. Cycle 3: (55X) Step 1: 95.0 °C for 10 s. Step 2: 60.0 °C for 1 min. cDNA (1 μL) was used in a total reaction volume of 20 μL for each reaction. Primers for rat *NGF* (Catalog # RN01533872) and *GAPDH* used in the TaqMan assay (RN9999916) were obtained from Applied Biosystems (Grand Island, NY, USA). Mouse *Oprd1* and rat *GAPDH* primers for SYBR Green are given in Table 1.

### 4.10. Agarose Gel Electrophoresis

PCR products were run on a 2% or 3% agarose gel for 40–45 min at 96 V and stained with ethidium bromide (0.5 μg/mL). Pictures were taken using a KODAK EDAS 290 High Performance UV transilluminator (Rochester, NY, USA).

### 4.11. Quantification of Gel Data

Gel bands were quantified with the Quantity One Basic Software (Basic Version, Bio-Rad, Hercules, CA, USA) using local background subtraction.

### 4.12. Statistical Analysis

Statistical differences were determined by ANOVA using StatView^®^ (version 5, SAS Institute Inc., Gary, NC, USA) for Windows. Differences were considered to be significant (* *p* < 0.05). The graphs were made using KaleidaGraph, version 4.03 (Synergy software, Reading, PA, USA, 2008).

## 5. Conclusions

Both opioid signaling and neurotrophic factor signaling have played an important role in neuroprotection and differentiation in the nervous system. Little is known about whether the crosstalk between these two signaling pathways will affect neuroprotection and differentiation. In this cell model study, our data together strongly suggest that delta opioid peptide DADLE acts through the NGF-induced functional G protein-coupled Oprd1 to provide its neuroprotective and differentiating effects at least in part by regulating survival and differentiating MAPK and PI3K/Akt signaling pathways in NGF-responsive rodent neuronal cells. Our data together with others’ studies also suggest that delta opioid agonists may be a potential lead compound for developing the NGF signaling activators and such NGF activators may be further developed for the prevention and treatment of NGF or other neurotrophic factor-dependent neurological disorders.

## Supplementary Information



## Figures and Tables

**Table 1:** Primer sequences used for PCR amplification.

Gene	Primer sequences
Rat *NGF* (115 bp)	forward: 5′ GCAGTGCCCCTGCTGAACCA 3′ reverse 5′ AAACAGCACGCGGGGTGAAC 3′
Rat *Oprd1* (110 bp)	forward 5′TACACTAAGCTGAAGACGGC 3′ reverse 5′TTTCCATCAGGTACTTGGC 3′
Rat *GAPDH* (119 bp)	forward 5′ GAAGGGCTCATGACCACAGT 3′ reverse 5′ GGATGCAGGGATGATGTTCT 3′
Mouse *Oprd1* (115 bp)	forward 5′ CCATCACCGCGCTCTACTC 3′ reverse 5′ GTACTTGGCGCTCTGGAAGG 3′

**Table 2:** Catalog ID and sequences of the 3 siRNA duplexes against rat *Oprd1* gene and that of the scrambled purchased from Integrated DNA Technologies (IDT).

Catalog ID	Duplex sequences
	
RNC.RNAI.N012617.8.1	5′ AGCUGAUCAACAUAUGCAUCUGGGT 3′
	3′ GUUCGACUAGUUGUAUACGUAGACCCA 5′

RNC.RNAI.N012617.8.2	5′ CAUUGGGACAGCUAGAAUAGGGCCC 3′
	3′ UGGUAACCCUGUCGAUCUUAUCCCGGG 5′

RNC.RNAI.N012617.8.3	5′ GGAAUCGUCCGGUACACUAAGCUGA 3′
	3′ AACCUUAGCAGGCCAUGUGAUUCGACU 5′

Scrambled duplex	5′ CUUCCUCUCUUUCUCUCCCUUGUGA 3′
	5′ UCACAAGGGAGAGAAAGAGAGGAAGGA 3′

**Table 3:** Catalog ID and sequences of the 3 siRNA duplexes against mouse *Oprd1* gene and that of the scrambled purchased from Integrated DNA Technologies (IDT).

Catalog ID	Duplex sequences
MMC.RNAI.N013622.12.4	5′ AGCUGAUCAAUAUAUGCAUCUGGGT 3′
	5′ ACCCAGAUGCAUAUAUUGAUCAGCUUG 3′

MMC.RNAI.N013622.12.5	5′ ACGUUGGAGAAGAGUCAAAGUUCTC 3′
	5′ GAGAACUUUGACUCUUCUCCAACGUUG 3′

MMC.RNAI.N013622.12.10	5′ GCAGUCAAUCUAAUGCUUUCCAACA 3′
	5′ UGUUGGAAAGCAUUAGAUUGACUGCGA 3′

Scrambled duplex	5′ CUUCCUCUCUUUCUCUCCCUUGUGA 3′
	5′ UCACAAGGGAGAGAAAGAGAGGAAGGA 3′
