# Supplementary material for: Crosstalk between Delta Opioid Receptor and Nerve Growth Factor Signaling Modulates Neuroprotection and Differentiation in Rodent Cell Models
Source: Int J Mol Sci. 2013 Oct 21;14(10):21114–39. doi: 10.3390/ijms141021114 (PMC3821661; doi:10.3390/ijms141021114)

Supplementary Information

Table S1. Sequencing results of PCR products

Gene name	Sequence obtained	BLASTn result
Rat *NGF*	GCGTGCTGTTTAGCACCCAGCCTCC ACCCACCTCTTCGGACACTC	Rat beta-nerve growth factor (NGF) gene, last exon. M36589.1 Expect-1e-10 Identity-100%
Rat *Oprd1*	GGCCACCAGCACACTGCCCTTCCAG AGCGCCAAGTACCTGATGGAAAA	Rattus norvegicus opioid receptor, delta 1 (Oprd1), mRNA. NM_012617.1 Expect-6e-17 Identity-100%
Mouse *Oprd1*	TGCTCGTCATGTTTGGCATCGTCCG GTACACCAAATTGAAACCGCCACC AACATCTACATCTTCAATCTGGCTT TGGCTGATGCGCTGGCCACCAGCA CGCTGCCC	Mus musculus opioid receptor, delta 1 (Oprd1), mRNA. NM_013622.3 Expect-7e-45 Identity-99%

Figure S1. RT-PCR analysis of time course of 10 nM DADLE treatment on endogenous *NGF* expression in PC12h cells. PC12h cells were treated simultaneously with 100 ng/mL NGF and 10 nM DADLE for varied times. After 72 h the total RNA was extracted and RT-PCR was carried out. (**A**) Induction of *NGF* mRNA after DADLE treatment in NGF stimulated PC12 h cells; (**B**) Relative optical density of NGF RT-PCR product with or without the DADLE treatment for varied times. Data is from one experiment with average of 3 PCR runs.

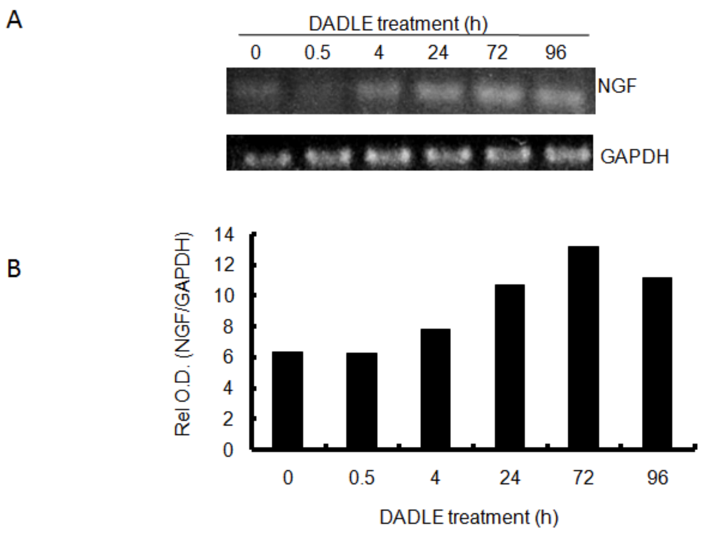

Figure S2. Effect of DADLE on the number of neurites normalized to the total cells and length of neurites normalized to the total neurites in PC12h cells. PC12h cells were cultured with 100 ng/mL NGF, with or without (control) 10 nM DADLE (40,000 cells per 35mm tissue culture dish). After 72 h of treatment, random pictures were taken (10 from each dish). Representative pictures of differentiating PC12h cells: (**A**) NGF treated only (control); (**B**) NGF + DADLE treated; (**C**) NGF + LY treated; (**D**) NGF + LY + DADLE treated; (**E**) NGF + PD treated; (**F**) NGF + PD + DADLE treated; (**G**) NGF + naltrindole treated; (**H**) NGF + naltrindole + DADLE treated.

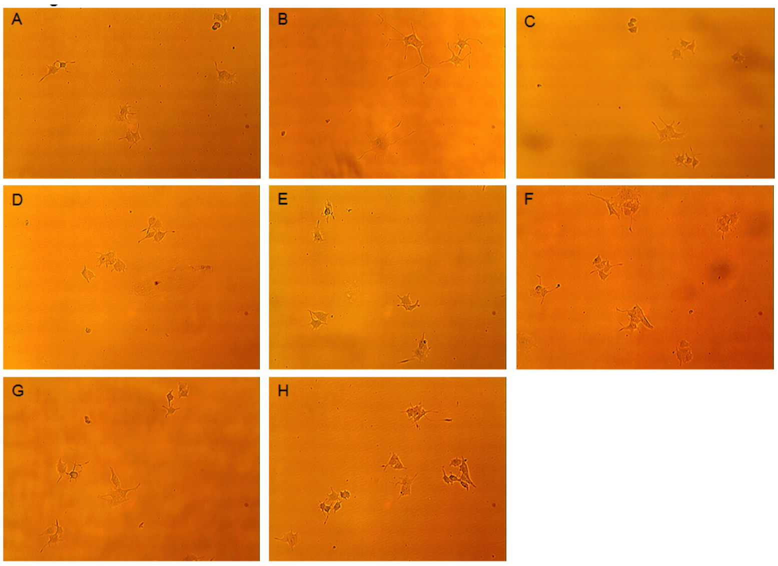

Figure S3. RT-PCR analysis of mouse *Oprd1* gene expression in F11 cells. F11 cells were cultured for 72 h in 3 different conditions, 0.1% serum containing medium, 0.1% serum medium + 0.5 mM db-cAMP, and 0.1% serum medium + 0.5 mM db-cAMP + 50 ng/mL NGF. After 72 h the total RNA was extracted and RT-PCR was carried out. (**A**) *Oprd1* cDNA expression; (**B**) Relative optical density of *Oprd1* RT-PCR product. Data are expressed as average of 2 independent experiments.

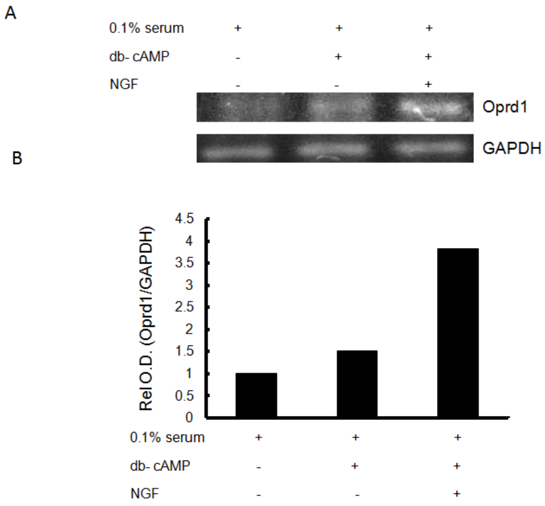

Figure S4. Effect of DADLE on time-dependent phosphorylation of MAPK and Akt in F 11 cells. F11 cells were differentiated with 0.5 mM db-cAMP in the presence or absence of 50 ng/mL NGF for 72 h. DADLE (1 µM) was treated for varied times. After a total of 72 h differentiation, the cells were harvested for total lysate and Western blotting was carried out for p-Akt (Ser 473), p-MAPK, total MAPK, total Akt, and beta actin as described in Materials and Methods. (**A**) Immunoblot showing DADLE induced phosphorylation of Akt and MAPK; (**B**) Semi-quantification of p-Akt normalized to total Akt; (**C**) Semi-quantification of p-MAPK normalized to total MAPK. Data is from one experiment.

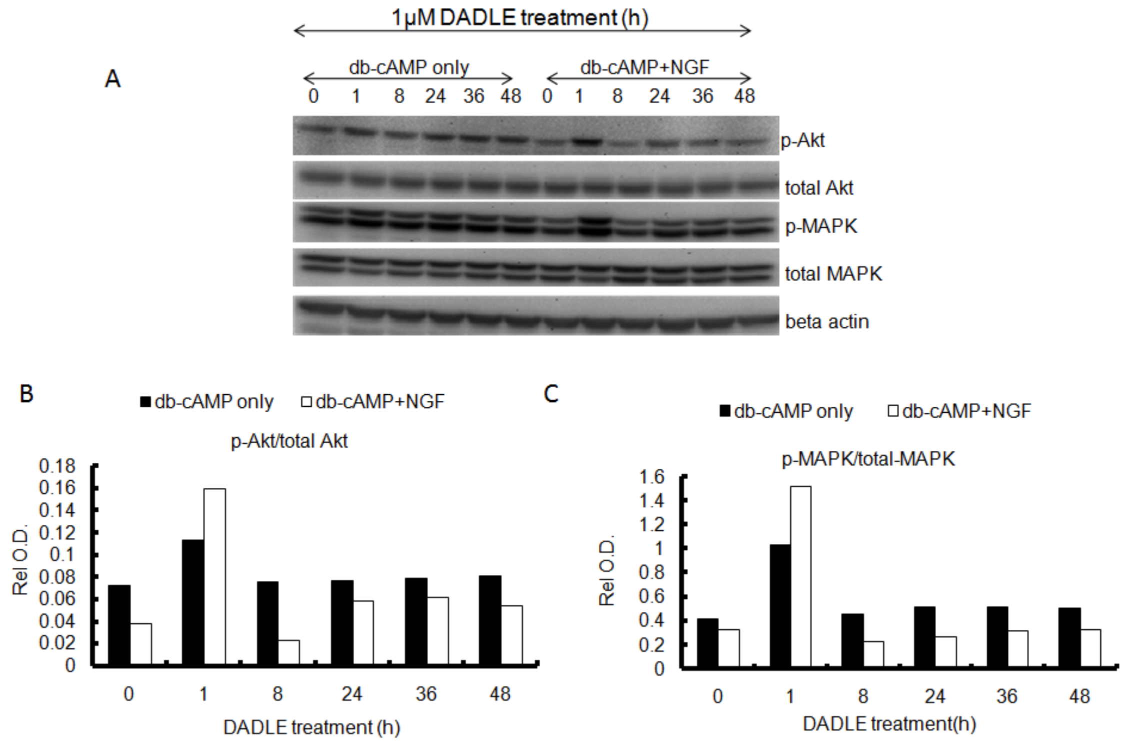

Supplement: Supplementary file 1 [file ijms-14-21114-s001.pdf]
